# Our double valve replacement strategy in a case with pectus carinatum deformity

**DOI:** 10.1186/1749-8090-8-S1-P21

**Published:** 2013-09-11

**Authors:** L Yilik, K Ergunes, U Yetkin, I Peker, I Yurekli, E Celik, A Gurbuz

**Affiliations:** 1 Department of Cardiovascular Surgery, Izmir Katip Celebi University Ataturk Training and Research Hospital, Izmir, Turkey

## Background

Pectus carinatum is a deformity of the chest characterized by a protrusion of the sternum and ribs. It develops as a result of an overgrowth of cartilage causing the sternum to protrude forward. It is detected most commonly during pubertal growth spurt.

## Methods

Our case was a 63-year-old male. An elective umbilical hernia repair was being planned. During the investigations due to shortness of breath, transthoracic echocardiography (TTE) revealed severe aortic and mitral regurgitation. Left ventricular end-diastolic and end-systolic diameters were measured as 72 and 48 mm, respectively. Left and right atrial diameters were measured as 56 and 55 mm, respectively. Pulmonary arterial pressure was calculated as 35 mm Hg and left ventricular ejection fraction was identified as 60%. Coronary angiography detected no significant stenoses and confirmed aortic and mitral regurgitation with an ejection fraction of 50%. Our case was consulted with Department of Pulmonary Diseases due to dyspnea and moderate pectus carinatum. Fibrotic lesions and pleural thickenings in the upper zones of both hemithoraces were interpreted as sequelae of tuberculosis. Pulmonary function tests were normal. Elective open heart surgery was planned.

## Results

After standard median sternotomy, aortic valve was replaced with 23 mm St. Jude mechanical valve and mitral valve was replaced with 31 mm St. Jude mechanical valve. Left atrial appendix was sutured internally due to atrial fibrillation to avoid embolization and posterior leaflet of the mitral valve was preserved. Postoperative period was event-free.

## Conclusions

Most cases with pectus carinatum are asymptomatic as in our case. Psychological and cosmetic disturbances may be observed as well as respiratory tract infections and exertional dyspnea. We think that technically challenging correction surgery should be avoided if patients are asymptomatic, particularly after completion of calcification process of the bony structures.

